# Infant and preschool attachment, continuity and relationship to caregiving sensitivity: findings from a new population‐based Australian cohort

**DOI:** 10.1111/jcpp.13865

**Published:** 2023-07-27

**Authors:** Jennifer E. McIntosh, Jessica Opie, Christopher J. Greenwood, Anna Booth, Evelyn Tan, Felicity Painter, Mariel Messer, Jacqui A. Macdonald, Primrose Letcher, Craig A. Olsson, ATPG3 MAC Lab and Consortium

**Affiliations:** ^1^ The Bouverie Centre, School of Psychology and Public Health La Trobe University Bundoora VIC Australia; ^2^ Centre for Social and Early Emotional Development, School of Psychology, Faculty of Health Deakin University Geelong VIC Australia; ^3^ Centre for Adolescent Health Murdoch Children's Research Institute Parkville VIC Australia; ^4^ Department of Paediatrics, Royal Children's Hospital The University of Melbourne Parkville VIC Australia; ^5^ Centre for Evidence and Implementation – Global Singapore Singapore

**Keywords:** Attachment, caregiving, proportions, cohort, social–emotional, infant, preschool

## Abstract

**Background:**

Here, we report new prevalence and temporal stability data for child attachment and parental caregiving behaviour, from infancy (1 year) to preschool (4 years).

**Methods:**

Attachment (SSP) and caregiving data (MBQS) were from observations of parents and their infants and preschoolers, who represent the third generation of participants within an Australian longitudinal cohort.

**Results:**

At 1 year (*n* = 314 dyads) and at 4 years (*n* = 368 dyads), proportions assessed secure were 59% and 71%, respectively. Proportions assessed avoidant were 15% and 11%; ambivalent 9% and 6%, and disorganised 17% and 12%, at 1 and 4 years. Continuity of attachment pattern was highest for the infant secure group. Of dyads initially classified disorganised in infancy, 36% remained so at the preschool assessment. Attachment and caregiving continuities across the infancy–preschool period were highest for the stable secure attachment group and lowest for the stable insecure attachment group. Loss of secure attachment to mother by age 4 years correlated with decreased maternal caregiving sensitivity, and acquisition of secure status by age 4 was associated with increased maternal sensitivity. We found no difference in caregiving sensitivity scores for mothers and fathers for female and male preschool children.

**Conclusions:**

The contemporary infant and preschool attachment proportions we report here closely mirror the patterns of those reported in prior decades, with an inclination towards secure base relationships. Our findings alert practitioners anew to the responsiveness of early attachment status to change in caregiving responsiveness and support ongoing investment in early identification of disorganised attachment.

## Introduction

Understanding social and emotional growth across early childhood is key to informing preventative and early remedial public health and clinical initiatives. Secure attachment and its continuity across the infancy–preschool period is one significant behavioural indicator of socio‐emotional adjustment (McIntosh et al., 2021). Secure behavioural patterns suggest the infant's ongoing assurance that their emotional states can be recognised by and co‐regulated with their caregiver. The attendant positive developmental cascade is now well documented. This includes optimal impacts on emerging brain structures and circuitry associated with regulatory capacity and a lasting positive developmental legacy for social and emotional functioning (Feldman, 2017; Granqvist et al., 2017; Madigan, Atkinson, Laurin, & Benoit, 2013).

Insecure, and specifically disorganised attachment relationships, have a clear association with socio‐emotional difficulties, particularly at the intersection of converging caregiving risk factors (Granqvist et al., 2017; Groh, Fearon, Ijzendoorn, Bakermans‐Kranenburg, & Roisman, 2017). Disorganised attachment behaviours convey a significant level of confusion in the infant's trust of the parent that, if sustained can over time, tend towards a pattern of controlling behaviour in preschool. Relative to the organised insecure categories (avoidant or ambivalent), disorganised attachment is associated with greater risk for poorer cognitive and socio‐emotional outcomes in middle childhood, later dissociative and borderline personality symptoms and suicidal ideation in adolescence and early adulthood (Lyons‐Ruth, 2008; O'Connor, Bureau, McCartney, & Lyons‐Ruth, 2011; van Ijzendoorn, Schuengel, & Bakermans‐Kranenburg, 1999).

While studies of infant and preschool attachment continue at pace in clinically indicated populations (e.g. McIntosh, Tan, Levendosky, & Holtzworth‐Munroe, 2021; Śliwerski, Kossakowska, Jarecka, Świtalska, & Bielawska‐Batorowicz, 2020), normative community estimates remain rare. This is perhaps of little surprise, given the resource‐intensive nature of reliable assessments of attachment, which depend on time‐consuming and expensive observations under set conditions, with specialist micro‐coding of child–parent interaction (Spies & Duschinsky, 2021).

### Attachment measurement and classification

To date, the gold‐standard method for assessing attachment in infants remains the Strange Situation Procedure (SSP) using the Ainsworth scales for interactive coding of attachment (Ainsworth et al., 1978) and the Main and Solomon (1990) indices for disorganisation and disorientation. With their advanced language and interpersonal skills, attachment behaviours in preschoolers present differently from those of infants, and a modified SSP assessment is used to reflect these developmental advances of the preschooler (Cassidy & Marvin, 1992).

Within both infant and preschool classification systems, secure attachment behaviours are characterised by confident and clear use by the young child of primary attachment strategies in times of stress, specifically seeking and maintaining contact with the caregiver for as long as needed to restore homeostatic balance following challenge or stress (Ainsworth et al., 1978; George & Solomon, 2008). In contrast, insecure attachments are classified as either organised (avoidant, resistant) or disorganised. Avoidant attachment patterns are characterised by the child's initiation of physical and/or psychological distance from their caregivers at peak times of need, with low or no seeking of contact, thought to reflect adaptation to their caregiver's minimisation of negative affective states (George & Solomon, 2008). The hallmark of resistant attachment patterns is amplification of attachment behaviour by both seeking and resisting contact with the caregiver when stressed, thought to be responsive to inconsistent caregiver response (George & Solomon, 2008).

Disorganised attachment relationships lack any of the above clear patterns. They are instead typified by moments of odd and mistimed behaviours in the young child during interaction with the caregiver. They appear incoherent in the context of the infant's stress state, reflecting what some regard as a collapse of the infant's attachment system (Duschinsky & Solomon, 2017; Main & Solomon, 1990). In the preschool years, the pattern is characterised by the child's controlling behaviours within what is regarded as a role‐reversed interaction with their caregiver (Cassidy & Marvin, 1992).

### Parent caregiving sensitivity and associations with offspring attachment

Strong replicated evidence continues to show the centrality of parental sensitivity in shaping offspring attachment patterns (e.g. Hawkins, Madigan, Moran, & Pederson, 2015; Verhage et al., 2016), as well as the contextual importance to the caregiving system of material and social support in the face of normative parenting stress (Booth, Macdonald, & Youssef, 2018).

From an early mother‐centric basis, Bowlby (e.g. Bowlby, 1988) came to emphasise the primary evolutionary function of the attachment system as keeping the child connected to one or more caregivers, whose balanced and coordinated attention contributed to the young child's secure base. Contemporary research embraces early attachment relationships as a distributive network (Dagan & Sagi‐Schwartz, 2021), including (but not limited to) the father–infant relationship (van Bakel & Hall, 2020). Yet as we show in the next section, inclusion of observations beyond the mother–child dyad in population‐based cohorts (child–father or non‐binary) remains rare.

### Continuity of attachment between infancy and preschool

A trend towards security over time in non‐clinical populations was noted in an early paper by Waters and Valenzuela (1999). Building upon prior research (Fraley, 2002; Pinquart, Feußner, & Ahnert, 2013; van Ijzendoorn et al., 1999), a recent meta‐analysis (Opie et al., 2021) summarised several decades of research on SSP‐based attachment continuity data across infancy to school entry period, and across 15 studies (*k* = 19), showed strong stability in secure attachments with 65% of securely attached infants also secure by the preschool years. This compared with 37%, 44% and 37% who transitioned to security from avoidant, resistant and disorganised, respectively, across the same period. Relatively less is known about temporal instability and change in parental caregiving behaviours in community samples, limiting developmental knowledge of one of the main drivers of attachment security in infant offspring.

### Bench‐marking attachment and caregiving proportions across the decades

Parenting roles and day‐to‐day availability have evolved in Western countries with increased maternal employment outside the home (Brooks‐Gunn, Han, & Waldfogel, 2010; Kim & Wickrama, 2021), growing father involvement in day‐to‐day care (Cabrera, 2020) and expanded use of early child care (Belsky et al., 2007; Hazen, Allen, Christopher, Umemura, & Jacobvitz, 2015; Vandell, Belsky, Burchinal, Steinberg, & Vandergrift, 2010). In the face of these changing societal influences, conceivably, as with childhood mental health trajectories (Sellers et al., 2019), early childhood attachment trends might be expected to alter across the decades. However, there has been no impact analysis of these broader societal changes on infant/child attachment and parent caregiving behaviours.

A recent systematic search (Opie et al., 2021) shows that in community samples (non‐clinical) of *N* ≥ 100, there has been seemingly little shift in infant or preschool attachment proportions by category or by parent gender since the first published findings (Ainsworth et al., 1978). Across these early childhood intervals, grouped findings suggest a small proportionate decrease in secure attachment status from infancy (64%) to preschool (59%), although within−study equivalence testing from the NICHD Early Child Care Research Network (2001) sample showed estimates were similar across the ages.

As another reference point for our current caregiving findings, we re‐examined community sample estimates of caregiving sensitivity estimates by SSP attachment classification from recent work by our team (Booth et al., 2018), utilising the Maternal Behaviour Q‐Sort (MBQS; Pederson et al., 1990; Pederson, Bailey, Tarabulsy, Bento, & Moran, 2014). Three studies report maternal MBQS scores against SSP status (Behrens, Parker, & Haltigan, 2011; Morley, Moran, Pederson, Bento, & Bailey, 2010; O'Connor et al., 2009). On the MBQS scale of −1 to +1 (−1 being extreme insensitivity and +1 being maximum sensitivity), means for the secure attachment group ranged from .55 to .75, and for insecure group, ranges were .05 to .48. Samples were small (*n* = 49–74 dyads). We found no studies including fathers.

### Aims of the current study

We aimed to describe Australia's first population‐level data on infant and preschool attachment behaviour, and parent caregiving sensitivity, collected between 2015 and 2020 in a large ongoing intergenerational study of social and emotional development, The Australian Generation 3 Study (ATPG3; detailed in a subsequent section). Specifically, we aimed to (a) estimate the prevalence and temporal stability of attachment classification from infancy to preschool in the ATPG3 sample, and (b) estimate the prevalence and temporal stability of caregiving behaviours from infancy to preschool in the ATPG3 sample. We later consider these estimates from our contemporary Australian cohort against meta‐analytic proportions from earlier international community cohorts.

## Methods

### Participants

Participants were infant–parent dyads (*n* = 314), and preschooler–parent dyads (*n* = 368) from a nested observational study of attachment, drawn from a large Australian intergenerational study of social and emotional development, the Australian Temperament Project Generation 3 Study (Olsson et al., 2022). The cohort commenced in 1983 as a study of temperament, with recruitment of Generation 1 (G1) parents 4–8 months after the birth of the study child (Generation 2: G2). In 1983, 67 Local Government Areas (LGAs) in the state of Victoria (urban and rural) were randomly selected based on Australian Bureau of Statistics, to provide a representative community sample (Sanson, Prior, & Oberklaid, 1985). The initial cohort comprised 2,443 G2 infants. The study has since tracked the social–emotional development of the main G2 cohort across three decades, with 15 waves of preconception survey data collection on temperament, internalising symptoms (i.e. depression and anxiety) and externalising behaviour (i.e. antisocial behaviour and substance use) as well as various measures of positive development and sociodemographic factors (Vassallo & Sanson, 2013).

The Generation 3 Study (G3 Offspring) began in 2011,^1^ recruiting existing ATPG2 participants and their infants through a systematic screening process yielding a sample of 1,167 G3 offspring born to 703 G2 parents. To date, G2 participants have been assessed via computer assisted telephone interviews and/or web surveys, in the third trimester of pregnancy, at 8 weeks postpartum, and at 1 and 4 years of age. A nested sample within this Generation 3 cohort were recruited to two observational assessments of attachment and caregiving, when the G3 child turned 1 and 4 years of age (Life@1 and Life@4 assessments respectively). Whether or not they had participated in the ATP cohort study, the parent who identified as the primary carer was subsequently invited with their infant/s into the Life@1 observational studies. The dominant mother sample reflects that requirement. Some parents participated in the infant and/or preschool‐SSP with more than one G3 child. As summarised in Table 1, the Life@1 sample comprised 314 infant–parent dyads (249 parents), with 235 ATPG2 mothers, 32 ATPG2 fathers and 47 non‐ATP mothers (no non‐ATP fathers).

**Table 1:** Demographic profile of the nested attachment sample

	Life@1	Life@4
Participants		
*N* child–parent dyads	314 249 parents, 312 children^b^	368 229 parents, 275 children^b^
SSP at both 1 and 4 years	166 dyads	
SSP with both parents	93 children	
Parent demographics		
Parent gender	282 Mothers^a^; 32 Fathers	253 Mothers; 115 Fathers
Parent mean age (years)	33.7 (33.6 Mo; 34.7 Fa)	35.3 (35.2 Mo; 35.6 Fa).
Parents married/DeFacto	87%	91%
Child demographics		
Child sex	169 females (54%); 143 males (46%)	147 females (53%); 128 males (47%)
Child mean age	1.2 years	4.3 years
Child first born	47%	55%
Child in any non‐family day care	61%	34%

^a^ The Life@1 parent was the self‐selected primary caregiver for the infant.

^b^ Numbers reflect parents who participated in the infant and/or preschool‐SSP with more than one G3 child over the years of the study.

Cohort retention strategies were utilised (Teague et al., 2018) in‐between data collection waves. Of all 249 parents who participated in the Life@1 observation, 166 parents (67%; 161 parent–child dyads) returned to participate in Life@4. Additionally, we recruited mother/father pairs to the study, whether or not they had participated in the first attachment observation. The final nested Life@4 sample comprised 368 child–parent dyads (229 parents, 275 children), with 163 ATPG2 mothers and 90 non‐ATP mothers (*N* = 253 mothers), and 112 ATPG2 fathers and three non‐ATP fathers (*N* = 115 fathers). 93 children had both mother and father participation. All data collection was conducted between April 2015 and April 2020, with data collection ceasing due to Australian government‐imposed restrictions related to the COVID‐19 pandemic.

All G2 participants were born in Australia and 82% reported year 12 or greater education. The G2 parents were predominantly married or in a de facto partnership when both the Life@1 and the Life@4 assessments were conducted (87% and 91%, respectively). Twin siblings comprised 4% of the infant sample (*n* = 6 twin pairs) and 3% of the preschool sample (*n* = 4 twin pairs).

Given the representativeness of the initial ATP recruitment (Sanson et al., 1985), to assess bias due to attrition, parents in the observational study were compared on characteristics collected at baseline (1983, at 4–8 months), including the ATP participants (G2) sex, difficult temperament, and behaviour problems, as well as their parent's (G1) education and country of birth. Overall, attrition analyses show the original sample retained since 1983 (and screened for the ATPG3 study) was less diverse with respect to G1 education and country of birth (Olsson et al., 2022). Compared with the full ATPG3 sample, those who participated in the 1‐year SSP were similar on baseline characteristics, although included fewer males. Similarly, those who participated in the 4‐year SSP were similar on baseline characteristics, although included a greater proportion of families with G1 fathers with non‐Australian births. In summary, the SSP samples were primarily less diverse in terms of G1 education and country of birth (and G2 participant sex for the 1‐year SSP) in comparison with the representative sample in 1983.

### Assessment procedures

#### Life@1 observational assessment

Life@1 assessments were conducted when G3 infants were aged between 12 and 18 months, at one of three city and regional sites within the ATP‐G3 Attachment and Caregiving (MAC) Lab at the Royal Children's Hospital, Melbourne. The filmed observations were typically 60 min in duration. Assessments were conducted by two to three researchers extensively trained in the SSP administration. G2 parents and their G3 infants completed a 10‐min orientation in the waiting room when free interaction between parent and infant was observed by the researchers. This was followed by the SSP in a novel playroom, as per the original eight‐episode protocol (Ainsworth et al., 1978; Ainsworth et al., 2015). Separations were shortened to 1 min if the child was distressed. Immediately before and following the SSP, 5 min of free play occurred, followed by a 15‐min collection of a bio‐sample of infant saliva in which the parent assisted a research assistant with swabbing and with height and weight measurement (noting bio‐metric data were beyond the scope of this manuscript). Parents were reimbursed for their time with a gift card, and infants given a suitable gift. Two fixed cameras recorded the events occurring in the playroom.

#### Life@4 observational assessment

When the G3 child was aged 4 years, G2 parents were again invited to visit the MAC Lab. This study additionally focused on father–child attachment. Therefore, two Life@4 observational sessions for each G3 child were conducted where possible, the first with the ATPG2 participant parent (whether G2 mother or G2 father) and the second with the non‐ATP parent when that parent had also participated in the Life@1 assessment. To reduce recall bias for the G3 child, each assessment was separated by 8–10 weeks, different toys were used and a different person played the role of stranger, and where possible, a different room or location was used. If the G3 child had only one parent or only one parent who wished to participate, these dyads completed only the first session.

Each Life@4 filmed observation was around 90 min in duration. A 10‐min observation in the waiting room occurred (not filmed) while general instructions were given, and pre‐session toileting took place. For all assessments, the filmed Life@4 visit began with the seven‐episode Preschool‐SSP (PS‐SSP) as per the original protocol (Cassidy & Marvin, 1992). Separations were shortened to 1 min if the child was very distressed. After the PS‐SSP, dyads completed a further 5 min extended play period, then a 15‐min compliance activity in the form of a pack‐up task, and a shared drawing cooperation task. The nature of these tasks was changed across sessions when both parents were involved in the study. The dyad was also filmed during saliva swab DNA collection, room pack‐up, task transition and instructions (20 min), and post‐session neuropsychological and cognitive assessment (30 min) and presentation to the parent of a gift card and to the child of a suitable gift. Measures of height/weight occurred in a separate room and were not filmed.

### Measurement and coding procedures and reliability

#### Infant attachment coding procedures

MAC Lab members completed full certification in four‐way Infant SSP coding with either or both the Minnesota Group and Dr Judith Solomon. Videotaped SSP data were analysed using the coding and decision‐making system for the organised categories by Ainsworth et al (2015) and for the disorganised category by Main and Solomon (1990). Dyads were classified at the two‐way (secure–insecure), two‐way (organised–disorganised), three‐way [secure (B), avoidant (A), ambivalent/resistant (C)] and four‐way levels (ABCD). At the four‐way level, classification of disorganised (D) relationship quality was coded on a nine‐point scale, across all interactions in the SSP which the parent was present. Additionally, assignment to one of eight organised sub‐categories or to the D category was made: B1 (secure sturdy), B2 (secure increasing proximity), B3 (secure prototypical), B4 (secure ambivalent); A1 (avoidant ignoring), A2 (avoidant neutral); C1 (ambivalent angry); C2 (ambivalent passive); D (disorganised).

To assess inter‐rater reliability, the first 179 SSP videos (57% of the total sample) were blind double‐coded, and disagreements resolved by conferencing or by referral to an expert (Judith Solomon). Inter‐rater agreement established prior to any external consultation was 87% (*κ* = 0.73) for the two‐way (secure/insecure) classification and 83% (*κ* = 0.74) for the four‐way (ABCD) classification.

#### Preschool attachment coding procedures

MAC Lab members trained with Prof. Marvin and completed full certification in the preschool‐SSP (PS‐SSP; Cassidy & Marvin, 1992), as the preferred research coding scheme for children aged between 30 and 54 months of age (Solomon & George, 2008). Coders were blind to the infant classification when the dyad had also participated in Life@1. Dyads were classified at the two‐way (secure‐insecure), two‐way (organised‐disorganised/insecure other), three‐way [secure (B), avoidant (A), ambivalent/resistant (C)] and four‐way levels (ABC and D/IO). Disorganised relationship quality was coded on all interactions in the SSP in which the parent was present. Additionally, assignment to one of 18 sub‐categories was made: B1 (secure reserved), B3 (secure prototypical), B4 (secure dependent, secure controlling, secure feisty or secure other); A1 (avoidant ignoring), A2 (avoidant neutral); C1 (ambivalent resistant); C2 (ambivalent immature); disorganised; controlling (caregiving, punitive, general); insecure/other (A/C avoidant and dependent, disengaged, inhibited/fearful or affectively dysregulated).

To assess inter‐rater reliability using Cassidy and Marvin's (1992) Preschool Attachment Coding System, the first 182 (49% of the total sample) SSP videos were blind double‐coded by reliable coders, and disagreements were resolved by conferencing or by referral to an expert (Professor Marvin). Inter‐rater agreement for classifications prior to conferencing was 88% (*κ* = 0.77) for the two‐way (secure/insecure) classification, and 85% (*κ* = 0.75) for the four‐way (ABCD) classification.

#### Caregiving sensitivity coding procedures (infancy)

Lead MAC Lab Coders were trained by Dr. Sandy Bento in the MBQS. Coding of Life@ 1 parental sensitivity was conducted by eight certified members of the MAC Lab, using the Mini‐Maternal Behaviour Q‐Sort‐V Revised Mini‐MBQS 25‐Item for Video Coding (Moran, 2009; Moran, Pederson, & Tarabulsy, 2011). Sensitivity was coded across all filmed interactions where the caregiver was present, as described above. Mini‐MBQS items are scored from −2 to +2, whereby lowest scores are given to items ‘least like’ the caregiver and highest scores to items ‘most like’ the caregiver. This generates an ‘unforced sort’ of item scores which is then ‘forced’ into a distribution of five equal groups of five items, as per conventions of q‐methodology (Herrington & Coogan, 2011). Each ‘sort’ is correlated with a criterion sort indicating the ‘prototypically sensitive’ parent. The resulting score can vary from −1 (least sensitive) to +1 (prototypically sensitive).

Inter‐rater reliability between coders prior to conferencing was identified using both percentage agreement and intraclass correlations (ICC). For percentage agreement, we adopted the Bento training standard that total MBQS scores be within .20 of one another. The inter‐rater reliability for infant MBQS data (*n* = 108) was 76% (ICC = .73).

#### Caregiving sensitivity coding procedures (preschool)

Parental sensitivity to preschoolers was coded across the set of filmed interactions described above. The available footage for coding totalled about 60 min given the parent was usually not present during the child's cognitive testing session. Sensitivity was assessed with the 25‐item Preschool Mini‐MBQS (PS‐MBQS; Tarabulsy et al., 2009). Coders were blind to the attachment classification. The PS‐MBQS has 25 items scored in the same way as the Infant MBQS. Item variations account for the child and dyad's developmental progressions, including advancements in secure base behaviours and enhanced emotional regulation skills. As with the Mini‐MBQS infancy scale, we report a score between −1 and +1, reflecting correlation of the forced score with a criterion sort indicating the ‘prototypically sensitive’ parent. The inter‐rater reliability for preschool MBQS data (*n* = 121) in this sample was 83% (ICC = .88).

### Statistical analyses

Data were analysed using Stata 17.0 (StataCorp, 2021). First, descriptive statistics for attachment classifications are presented as counts/proportions. Differences in attachment classifications between child sex and parent gender are examined using a Chi‐squared test. Second, descriptives for caregiving sensitivity are presented as means and standard deviations. Point‐biserial correlations were used to examine the relationship between MBQS scores and binary indicators of attachment (security vs. insecurity, not‐avoidant vs. avoidant, not‐resistant vs. resistant, organised vs. disorganised). To examine differences in the pattern of MBQS scores across attachment classifications for child and parent sex linear regression analyses were used. Specifically, MBQS scores are regressed onto attachment classification, a sex variable and the interaction between the two; with the interaction used to determine differences. Third, we examined the stability of attachment and caregiving sensitivity across 1‐year and 4‐year assessments. Attachment classification stability is examined descriptively and using Chi‐squared test and Cohen's Kappa (*κ*). Caregiving stability is examined using Pearson's *r*. Differences in the stability of caregiving sensitivity MBQS scores (1‐year, 4‐year, and difference scores) were regressed onto a four‐level attachment stability variable (secure–secure, secure–insecure, insecure–secure and insecure–insecure). To account for within parent (i.e. multiple children clustering), all regression models employed robust (clustered) standard errors which allow for intragroup correlation, relaxing the usual requirement that the observations be independent (Cameron & Miller, 2015).

## Results

### Contemporary Australian attachment proportions

#### Distribution of attachment classifications at 1 and 4 years

Tables 2 and 3, for the 1‐year and 4‐year samples, respectively, show the distributions across 4‐way (secure, avoidant, ambivalent, disorganised) and 2‐way (organised/disorganised, secure/insecure) main classifications, and relevant sub‐classifications, stratified by infant sex and parent gender. For both the 1‐year and 4‐year samples, patterns of 4‐way attachment classifications were not different across both infant sex (1‐year: *χ* ^2^ (3) = 2.61, *p* = .46; 4‐year: *χ* ^2^ (3) = 1.21, *p* = .75) and parent gender (1‐year: *χ* ^2^ (3) = 5.93, *p* = .12; 4‐year: *χ* ^2^ (3) = 3.67, *p* = .30). At 4 years, patterns of four‐way attachment classification were not different for children across assessments with both parents (*χ* ^2^ (3) = 2.72, *p* = .44; Tables 2 and 3).

**Table 2:** Infant attachment classifications in the ATP‐G3 Life@1 cohort

	Full sample (*n* = 314), *n* (%)	By infant sex		By parent gender	
		G3 Girls (*n* = 171), *n* (%)	G3 Boys (*n* = 143), *n* (%)	Mother (*n* = 282), *n* (%)	Father (*n* = 32), *n* (%)
Major classifications					
Secure (B)	185 (59)	102 (60)	83 (58)	160 (57)	25 (78)
Avoidant (A)	48 (15)	22 (13)	26 (18)	46 (16)	2 (6)
Ambivalent (C)	29 (9)	15 (9)	14 (10)	28 (10)	1 (3)
Disorganised (D)	52 (17)	32 (19)	20 (14)	48 (17)	4 (13)
Binary classifications					
Organised (B, A, C)	262 (83)	139 (81)	123 (86)	234 (83)	28 (88)
Insecure (A, C, D)	129 (41)	69 (40)	60 (42)	122 (43)	7 (22)
Organised attachment sub‐classifications					
B1 (Secure sturdy/robust)	49 (16)	30 (18)	19 (13)	41 (15)	8 (25)
B2 (Secure increasing proximity)	54 (17)	29 (17)	25 (17)	49 (17)	5 (16)
B3 (Secure prototypical)	35 (11)	18 (11)	17 (12)	28 (10)	7 (22)
B4 (Secure ambivalent)	47 (15)	25 (15)	22 (15)	42 (15)	5 (16)
A1 (Avoidant ignoring)	30 (10)	12 (7)	18 (13)	30 (11)	0 (0)
A2 (Avoidant neutral)	18 (6)	10 (6)	8 (6)	16 (6)	2 (6)
C1 (Ambivalent angry)	14 (4)	8 (5)	6 (4)	14 (5)	0 (0)
C2 (Ambivalent passive)	15 (5)	7 (4)	8 (6)	14 (5)	1 (3)

A = Avoidant; B = Secure; C = Ambivalent; D = Disorganised.

**Table 3:** Preschool attachment classifications in the ATP‐G3 Life@4 cohort

	Full sample (*n* = 368), *n* (%)	By child sex		By parent gender	
		G3 Girls (*n* = 193), *n* (%)	G3 Boys (*n* = 175), *n* (%)	Mother (*n* = 253), *n* (%)	Father (*n* = 115), *n* (%)
Major classifications					
Secure (B)	262 (71)	137 (71)	125 (71)	176 (70)	86 (75)
Avoidant (A)	41 (11)	19 (10)	22 (13)	27 (11)	14 (12)
Ambivalent (C)	22 (6)	12 (6)	10 (6)	15 (6)	7 (6)
Disorganised/insecure‐other (D/IO)	43 (12)	25 (13)	18 (10)	35 (14)	8 (7)
Binary classifications					
Organised (B, A, C)	325 (88)	168 (87)	157 (90)	218 (86)	107 (93)
Insecure (A, C, D/IO)	106 (29)	56 (29)	50 (29)	77 (30)	29 (25)
Sub‐classifications					
B1 (Secure Reserved)	73 (20)	34 (18)	39 (22)	51 (20)	22 (19)
B3 (Secure prototypical)	28 (8)	10 (5)	18 (10)	20 (8)	8 (7)
B4 (Secure dependent, controlling, feisty, other)	161 (44)	93 (48)	68 (39)	105 (42)	56 (49)
A1 (Avoidant ignoring)	9 (2)	2 (1)	7 (4)	6 (2)	3 (3)
A2 (Avoidant neutral)	32 (9)	17 (9)	15 (9)	21 (8)	11 (10)
C1 (Ambivalent resistant)	9 (2)	4 (2)	5 (3)	6 (2)	3 (3)
C2 (Ambivalent immature)	13 (4)	8 (4)	5 (3)	9 (4)	4 (3)
Disorganised	3 (1)	2 (1)	1 (1)	3 (1)	0 (0)
Controlling	5 (1)	5 (3)	0 (0)	3 (1)	2 (2)
Insecure/other	21 (6)	13 (7)	8 (5)	18 (7)	3 (3)
A/C (Avoidant & Dependent)	9 (2)	3 (2)	6 (3)	7 (3)	2 (2)
Disengaged	0 (0)	0 (0)	0 (0)	0 (0)	0 (0)
Inhibited fearful	0 (0)	0 (0)	0 (0)	0 (0)	0 (0)
Affectively dysregulated	5 (1)	2 (1)	3 (2)	4 (2)	1 (1)

A = Avoidant; B = Secure; C = Ambivalent; D = Disorganised; I/O‐Insecure‐Other.

#### Caregiving sensitivity by attachment classification at 1 and 4 years

Table 4 provides MBQS caregiving sensitivity (forced scores) means and standard deviations across attachment classifications, stratified by child sex and parent gender, at 1 and 4 years. MBQS scores were negatively correlated at 1 year with insecurity (*r* = −.69, *p* < .001), avoidant (*r* = −.18, *p* = .002), resistant (*r* = −.26, *p* < .001) and disorganised (*r* = −.53, *p* < .001) and at 4 years with Insecurity (*r* = −.71, *p* < .001), avoidant (*r* = −.26, *p* < .001), resistant (*r* = −.26, *p* < .001) and disorganised (*r* = −.55, *p* < .001).

Patterns of 1‐year MBQS scores across four‐way (secure, avoidant, ambivalent, disorganised) attachment classifications did not vary across child sex (interaction *p* = .53) but varied by parent gender (interaction *p* = .015), such that MBQS scores were lower for infants classified as resistant for mothers compared to fathers, although the small sample size for fathers of resistant infants (*n* = 1) makes comparisons difficult. Patterns of 4‐year MBQS scores across four‐way (secure, avoidant, ambivalent, disorganised) attachment classifications did not vary across child sex (interaction *p* = .86) or parent gender (interaction *p* = .91).

**Table 4:** MBQS by attachment classification

	Full sample Mean (SD)	Child sex		Parent gender	
		Girls Mean (SD)	Boys Mean (SD)	Mothers Mean (SD)	Fathers Mean (SD)
Infancy (1‐year)					
Secure (B) *n* = 185	0.59 (0.2)	0.60 (0.2)	0.57 (0.2)	0.60 (0.2)	0.52 (0.2)
Avoidant (A) *n* = 48	0.25 (0.3)	0.22 (0.3)	0.27 (0.3)	0.25 (0.3)	0.28 (0.1)
Ambivalent (C) *n* = 29	0.11 (0.2)	0.16 (0.3)	0.06 (0.2)	0.11 (0.2)	0.24 (.)
Disorganised (D) *n* = 52	−0.01 (0.3)	−0.04 (0.3)	0.02 (0.3)	−0.02 (0.3)	0.09 (0.5)
Organised (B, A, C) *n* = 262	0.47 (0.3)	0.49 (0.3)	0.45 (0.3)	0.47 (0.3)	0.49 (0.2)
Insecure (A, C, D) *n* = 129	0.11 (0.3)	0.09 (0.3)	0.14 (0.3)	0.11 (0.3)	0.17 (0.3)
Preschool (4‐year)					
Secure (B) *n* = 262	0.58 (0.2)	0.60 (0.2)	0.55 (0.2)	0.59 (0.2)	0.55 (0.2)
Avoidant (A) *n* = 41	0.23 (0.2)	0.27 (0.2)	0.18 (0.2)	0.25 (0.2)	0.18 (0.2)
Ambivalent (C) *n* = 22	0.13 (0.2)	0.12 (0.2)	0.14 (0.3)	0.13 (0.3)	0.13 (0.2)
Disorganised/insecure‐other (D/IO) *n* = 43	−0.01 (0.3)	0.02 (0.2)	−0.04 (0.4)	−0.01 (0.3)	−0.01 (0.3)
Organised (B, A, C) *n* = 325	0.50 (0.2)	0.53 (0.2)	0.47 (0.3)	0.52 (0.2)	0.47 (0.2)
Insecure (A, C, D/IO) *n* = 106	0.11 (0.3)	0.13 (0.2)	0.09 (0.3)	0.11 (0.3)	0.11 (0.2)

A = Avoidant; B = Secure; C = Ambivalent; D = Disorganised; I/O‐Insecure‐Other.

### Stability of attachment across infancy and preschool years

Tables 5 and 6 present the two‐way and four‐way stability of attachment classifications, for the infant and preschool samples, respectively.

At the two‐way level, observed attachment stability between infancy and preschool was highest for the organised group (90%), followed by the secure group (80%). Of attachments classified disorganised at infancy, a move to organisation was more common (52%) than stable disorganisation (40%). Of attachments classified secure in infancy, 20% moved to an insecure classification. For attachments classified insecure at infancy, a move to security was more common (60%) than stable insecurity (44%).

When examined at the four‐way level, highest attachment classification continuity between infancy and preschool was observed in the secure group (80%) followed by disorganisation (40%), avoidance (27%) and resistance (22%). Greatest movement was seen to preschool secure classification from infant resistance (61%), avoidance (57%) and disorganisation (52%). Loss of security was not common (20%) and when it occurred, was distributed across the insecure preschool classifications.

**Table 5:** Two‐way attachment stability contingency table

Infancy	Preschool		
	Secure	Insecure	Total
Secure	**70 (80%)**	18 (20%)	88
Insecure	41 (56%)	**32 (44%)**	73
Total	111	50	

	Organised	Disorganised	Total
Organised	**122 (90%)**	14 (10%)	136
Disorganised	15 (60%)	**10 (40%)**	25
Total	137	24	

Diagonal value in bold represent stable attachment classification; Secure/Insecure: *χ* ^2^ (1) = 10.12, *p* = .001; *κ* = 0.24; Organised/Disorganised: *χ* ^2^ (1) = 14.69, *p* < .001; *κ* = 0.30.

**Table 6:** Four‐way secure/avoidant/ambivalent/disorganised attachment stability contingency table

Infancy	Preschool				
	Secure	Avoidant	Resistant	Disorganised/I/O	Total
Secure	**70 (80%)**	6 (7%)	5 (6%)	7 (8%)	88
Avoidant	17 (57%)	**8 (27%)**	0 (0%)	5 (17%)	30
Resistant	11 (61%)	1 (6%)	**4 (22%)**	2 (11%)	18
Disorganised	13 (52%)	1 (4%)	1 (4%)	**10 (40%)**	25
Total	111	16	10	24	

Diagonal value in bold represent stable attachment classification; Secure/Avoidant/Resistant/Disorganised: *χ* ^2^ (9) = 37.02, *p* < .001; *κ* = 0.25.

### Stability of caregiving sensitivity

**Figure 1:**
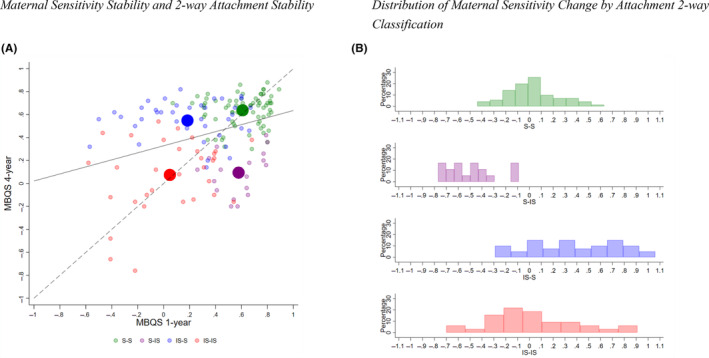
(A) Maternal sensitivity stability and two‐way attachment stability. (B) Distribution of maternal sensitivity change by attachment two‐way classification. S‐S = secure to secure, S‐IS = secure to insecure, IS‐S = insecure to secure, IS‐IS = insecure to insecure. The grey dashed line represents the line of perfect stability; solid black line represents line of stability in the current sample [Color figure can be viewed at wileyonlinelibrary.com]

A moderate positive correlation was observed between 1‐year and 4‐year MBQS scores (*r* = .35, *p* < .001), suggesting moderate stability. Figure 1A shows that MBQS scores were highest at both 1‐year and 4‐year assessments for those with stable secure attachments (1‐year: S‐S = base; S‐IS *b* = −0.03, *p* = .50; IS‐S *b* = −0.43, *p* < .001; IS‐IS *b* = −0.56, *p* < .001; and 4‐year: S‐S = base; S‐IS *b* = −0.54, *p* < .001; IS‐S *b* = −0.09, *p* = .004; IS‐IS *b* = −0.56, *p* < .001). In contrast, MBQS scores were lowest at both 1‐year and 4‐year assessments for those with stable insecure attachments (1‐year: IS‐IS = base; S‐S *b* = 0.56, *p* < .001; S‐IS *b* = 0.53, *p* < .001; IS‐S *b* = 0.14, *p* = .11; and 4‐year: IS‐IS = base; S‐S *b* = 0.56, *p* < .001; S‐IS *b* = 0.02, *p* = .78; IS‐S *b* = 0.47, *p* < .001). Figure 1B shows a pattern of change in MBQS scores for dyads who either lost attachment security or gained attachment security. MBQS scores had the least change for those with stable attachment classifications (S‐S = base; S‐IS *b* = −0.51, *p* < .001; IS‐S *b* = 0.34, *p* < .001; IS‐IS *b* = −0.00, *p* = .98).

## Discussion

These new community cohort data from Australia add to the existing literature on early life attachment and caregiving in three important ways. First, our contemporary findings provide current estimates for two, three and four‐way attachment classifications in community infant and preschool samples, for males or females. We found the frequency of secure attachments at age 4 years was higher than at infancy. Relative to historic cohort data (Opie et al., 2021), we observe little drift in these estimates across the decades. Second, continuity of attachment organisation between 1 and 4 years was high, at 92% (B, A, C) and 36% of the initial disorganised group. Continuous secure classification was 83%. Greatest movement was towards security, and loss of secure relationship quality was least common. Third, we found strong correlations between both attachment and caregiving continuities and discontinuities. Loss of secure attachment to mother by age 4 years correlated with decreased maternal caregiving sensitivity between assessment intervals, and conversely, acquisition of security by age 4 was positively associated with increased maternal sensitivity across the preschool years. In the preschool sample, there was no difference in caregiving sensitivity scores for mothers and fathers by attachment classification for infant females and males. We consider these findings in context, below and contrast them along the way to historic proportions.

### Attachment findings

#### Prevalence of security in infancy

We found 59% secure attachment, 15% avoidant, 9% ambivalent/resistant and 17% disorganised. Within groups, A1 (avoidant ignoring) was nearly twice as common as the A2 sub‐group (avoidant neutral). Sex differences were few in the major classifications, although infant girls were 1.5 times more likely to be assigned to the disorganised group.

#### Contrast to prior infant norms

Each infant estimate sits well within the range reported in historic proportions (Opie et al., 2021). Without equivalence testing, we can only note broad similarity to the large NICHD infant cohort of 1,060 Mother–infant dyads which reported 62% secure at 15 months, collected in the 2000–2010 decade. Proportions for the insecure A and C groups reflect prior estimates reported across decades. Our estimate for infant disorganised attachment may reflect a small advance on means reported in Opie et al. (2021). Overall lack of change in these estimates may be a greeted finding, suggesting minimal impact of a changing sociology of parenting practices. It may also raise the issue of how sensitive the SSP and sensitivity measures are to real change in parenting practices that has occurred across these decades. Given our study was not designed to examine these trends or their sociological associations, future research enquiry is needed.

#### Higher security proportion by 4 years and contrast to prior estimates

Overall, we see some key differences in our preschool cohort relative to our infant sample. We found 71% secure relationship classification at preschool, contrasted to 59% in the infant sample. The finding may reflect the older age of our preschool cohort which averaged 51 months relative to prior studies' average of 40 months (Opie et al., 2021). This additional year may allow parents a longer period of settling into family life, adjusting to return to work and coordinating caregiving across the child's extended attachment network.

#### Child attachment and parent gender

Estimates were not different across our preschool attachment classifications for mothers and fathers (253 mothers and 115 fathers, respectively), reflecting findings from prior cohorts (Opie et al., 2021). In the infant sample, mothers and fathers were equally likely to be assigned to disorganised dyads, but secure classifications were proportionally greater for father–infant dyads. However, given this sample comprised only the self‐nominated primary caregiver, disparity in mother and father sample sizes resulted (282 v 32 respectively), precluding analyses of gender differences.

Possible context for mother–father security differences at 1 year but not at 4 years includes employment demography. Australian national paid parental leave provides 18 weeks paid leave at the National Minimum Wage. Working partners are eligible to receive 2 weeks paid leave. A majority of mothers identify as the primary caregiver and return to work from infant age 11 months (Australian Bureau of Statistics, 2018; Baxter, 2008). With ATP‐G3 assessments occurring at 12 months years, it is possible that early adjustment to prolonged separation from mother may be reflected in lower secure rates with mothers, relative to the preschool findings. By the 4th birthday, in Australia 95% of children are enrolled in preschool for 15 hours or more per week (Australian Institute of Health and Welfare, 2022), and lengthy separations from parents are normative.

Future research is needed in this space, including consideration of the impact of rising quality standards for out‐of‐home care. For example, Australia's National Quality Framework (2021) provides regulatory guidelines for provision of skilled personalised care to young children. In turn, provision of effective attachment networks by age four may support continuity of child–parent attachment organisation, and indeed growth of security across early childhood.

### Attachment continuity findings

From infancy to preschool, we identified 92% stability of the organised group (B + A + C) and 36% in the disorganised group. Greatest movement was towards security, from infant avoidance (66%), resistance (63%) and disorganisation (48%). Loss of security was least common, and when occurring was mainly distributed to avoidant and disorganised preschool classifications. The general movement towards security is consistent with prior infancy–preschool attachment stability literature (Fish, 2004; Opie et al., 2021; Vice, 2004). Such results may support the non‐determinative nature of early attachment, consistent with Bowlby's first theorising (Bowlby, 1969).

Of concern though are our findings that 36% of mother–infant dyads initially classified disorganised were again classified disorganised at the preschool assessment. This means 6% of the entire sample were classified disorganised in attachment at both intervals. These are congruent with prior cohort data. Elsewhere, emphasis is given to the importance of not pathologizing a cross‐sectional classification of disorganised attachment as indicative of child psychopathology or maltreatment (Granqvist et al., 2017). However, few would argue that a continuous disorganisation pattern at both 1 year and 4 years presents a different developmental equation. There will be important lessons to learn from this group in time. In the interim, we suggest ‘continuously disorganised attachment’ beginnings warrant earliest detection, and close and compassionate attention by public health practitioners.

### Caregiving findings

Mean sensitivity scores in ATP‐G3 dyads later classified secure were 0.58 and 0.56 for infants and preschoolers, respectively, 0.22 and 0.28 in dyads later classified avoidant, and 0.07 and 0.17 in dyads later classified resistant/ambivalent. In both child age groups, the average score for caregiving in dyads later classified disorganised was 0.0. These findings accord closely with two Canadian community cohorts of adult mothers with the same measure (Morley et al., 2010; O'Connor et al., 2009). We suggest our findings consolidate evidence for the validity of the MBQS‐Mini as a measure of caregiving sensitivity in infancy. This is a practically important finding given the accessibility and relative ease of training in this method.

Practitioners may also find helpful our Life@4 findings of marked commonality of sensitivity for mothers and fathers across all attachment classifications, for female and male children. This supports the merits of observing the parental caregiving system as if it were universal rather than a gendered caregiving system, echoing Cassidy's view that ‘it is the nature of the interaction rather than the category of the individual that is important to the child’ (Cassidy, Jones, & Shaver, 2013, p. 1422).

### Caregiving and attachment continuity associations

#### Attachment and caregiving stabilities (maternal data)

We found positive correlations between the stability of maternal caregiving sensitivity and of child attachment relationship quality, strongest for the stable secure group. The same analysis for fathers was not possible given few fathers completed both Life@1 and Life@4 assessments, by virtue of the study design. Loss of secure attachment was clearly associated with decreased caregiving sensitivity over time, and conversely, acquisition of security by age 4 was associated with increased maternal sensitivity across the preschool years. The most scattered patterns were evident in the continuous insecure group, where MBQS change scores ranged from −0.7 to 0.9. These findings are consistent with a prior study of MBQS stability spanning a similar interval (Behrens, Parker, & Kulkofsky, 2014).

Our data show a growth in maternal caregiving sensitivity correlating with the infant's move towards secure attachment relationship quality by age 4 (Figure 1A). Given infant use of probabilistic inference of the likelihood of successful response to their needs (Cassidy et al., 2013), accruing stable information for these infants may have consolidated into a secure response to mother in the SSP by age 4 years. This conjures Waters and Valenzuela (1999, p. 271) note some decades ago, of the organism's ‘self‐righting tendencies’ in development and the likely increasing influence of the child's active efforts to elicit care. Practical implications of these findings are important and include translation of these developmental mechanisms into parent educational materials (e.g. Opie, Hooker, Gibson, & McIntosh, 2023).

### Strengths and limitations

Our cohort data have significant strengths with their origins in a representative community sample, large sample sizes, use of well validated, ‘gold‐standard’ observational tools, inclusion of child–mother and child–father attachment and caregiving observations. In general population cohorts, these attributes remain rare. Valid coding of the SSP and MBQS was a high priority in this study, as were strong efforts to standardise caregiving observations across study phases, and to recruit a significant father–child sample for the preschool study. Methodologically, our caregiving observations were far longer than those typically reported and covered an array of play and task‐focused activity between parent and child (Booth et al., 2018). Our correlation findings between attachment and caregiving at 1 and 4 years is rarely available in stability studies. The latter is useful information for evaluating validity and stability of the MBQS. To these ends, these new cohort data add clear value.

Selective attrition is a limitation with any prospective cohort study. While those participating in the observational assessments were broadly representative of the full G3 retained cohort (*n* = 1,701) on baseline characteristics (1983), the G3 sample did differ from the original sample (*n* = 2,443), with marginally higher rates of drop out in G1 parents who were non‐Australian born and with lower education levels (Olsson et al., 2022). Marginally higher economic security in our retained nested cohort may reflect marginally lower family stress levels, which could translate to slight gains in caregiving sensitivity based on previously reported meta‐analytic associations between disadvantage and caregiving (Atkinson et al., 2000; De Wolff & van Ijzendoorn, 1997).

Additionally, the ATPG3 sample included children born to participants aged 29–35 years, the peak reproductive years and it is possible prevalence and temporal stability estimates differ in children of younger and older parents. Replication in samples with a broader age of parental recruitment is required. We presented preliminary data on cross‐sectional associations between caregiving and offspring attachment, but do not model prospective, pre‐conception pathways or assess mediational pathways (genetics, socio‐economics, parent mental health, child cognitive development). These and other topics such as the relation between mother and father attachment and prospective associations with offspring well‐being are the subject of forthcoming studies.

Methodologically, a possible coding confound may be in the short overlapping interval in SSP and MBQS observations during opening and closing episodes of the SSP, each linked to extended free play. Even so, other interactions represented a large proportion of the caregiving observation, reducing the potential for a systematic confound. With resourcing, future research could re‐code the same footage contrasting scores from only non‐SSP time with those reported here. Finally, different interactions in Life@1 (orientation in the waiting room) and Life@4 (several tasks) may have biased the results, as certain tasks particularly the shared drawing activity for preschool dyads may have elicited more sensitive responses from parents than generic comforting of a baby in new surrounds.

## Conclusions

For Bowlby (1988, pp. 163–164), ‘No concept within the attachment framework is more central to developmental psychiatry than that of the secure base’. Given the now well‐established contributions of early relational health to later mental health, identifying wellness and difficulty in the development of trust remain key to health promotion and prevention pathways alike.

On these lines, the messages from our contemporary Australian cohort are encouraging, suggesting as they do a normative developmental inclination towards secure base relationships in the preschool years. Noteworthy and indeed heartening, our data show strong continuity of early secure attachment and a dominant tendency for movement from disorganisation and insecurity to security by age 4. Subsequent papers will explore antecedent and moderated pathways. Our findings underscore the highly responsive nature of early childhood attachment to change in caregiving responsiveness, for better and for worse.

We also identify an important minority, namely the 6% of our community sample showing continuous disorganisation from infancy to preschool. Given the prohibitive cost and viability problems of including gold‐standard attachment assessment within public health surveillance, a recent focus on identifying early indicators of infant relational health and training frontline workforces in their sensitive use holds promise (Ammitzbøll, Holstein, Wilms, Andersen, & Skovgaard, 2016; Clancy et al., 2020; McIntosh, Olsson, et al., 2021). The potential for decreased prevalence of later childhood and adolescent socio‐emotional health difficulties through investment of resources is clear. So too is the potential to learn from contemporary longitudinal community cohorts about the foundations of relational health.

## Endnote

1 Ethics approval from Royal Children's Hospital (Project No: 34185) under the project title “Australian Temperament Project Generation 3 Study”.
